# Gut Microbial Profile Changes in Patients with Gallbladder Stones after UDCA/CDCA Treatment

**DOI:** 10.3390/biomedicines11030777

**Published:** 2023-03-03

**Authors:** Jungnam Lee, Sohee Lee, Hanul Kim, Jaewoong Bae, Jin-Seok Park

**Affiliations:** 1 Department of Internal Medicine, Inha University Hospital, Inha University School of Medicine, Incheon 22332, Republic of Korea; 2 R&D Institute, BioEleven Co., Ltd., Seoul 06220, Republic of Korea

**Keywords:** GB stone, UDCA, CDCA, microbiome, Roseburia

## Abstract

**Background:** Ursodeoxycholic acid (UDCA) and chenodeoxycholic acid (CDCA) are used to treat patients with asymptomatic or mildly symptomatic gallstone disease. This study was conducted to evaluate the efficacy of gallbladder (GB) stone dissolution by UDCA/CDCA and the impact of treatment on gut microbial profiles. **Methods:** Fifteen treatment-naive patients with GB stones were initially included, but two dropped out during the treatment period. UDCA/CDCA was administered for 6 months. Abdominal ultrasonography was performed to evaluate response to treatment. In addition, fecal samples were collected before and after treatment for gut microbiome profiling. Then, 16S ribosomal RNA gene sequencing was carried out on fecal samples obtained before and after treatment, and results were compared with those of forty healthy controls. **Results:** Eight (62%) of the thirteen evaluable patients treated with UDCA/CDCA responded to treatment (four achieved complete GB stone resolution and four partial dissolution). Taxonomic compositions of fecal samples at the phylum level showed a significantly lower relative abundance of the *Proteobacteria* phylum in the pre-UDCA/CDCA group than in the healthy control group (*p* = 0.024). At the genus level, the relative abundances of five bacteria (*Faecalibacterium*, *Roseburia*, *Lachnospira*, *Streptococcus*, and *Alistipes*) differed in the control and pre-UDCA/CDCA group. Interestingly, the abundance of *Roseburia* was restored after 6 months of UDCA/CDCA treatment. **Conclusion:** Gut microbial dysbiosis was observed in GB stone patients and partially reversed by UDCA/CDCA treatment, which also effectively dissolved GB stones.

## 1. Introduction

Gallbladder (GB) stones are solid mixtures of calcium bilirubinate, cholesterol, and proteins, and are not uncommon in adults [1,2]. Around 10% of adults and 20% of individuals over the age of 65 have gallstones, and up to 20% of those with gallstones develop symptoms such as biliary colic or infections [2]. Furthermore, gallstone disease is reported to account for 13 to 50% of gastrointestinal diseases in Western countries, and because of its high prevalence gallstone disease is a socially significant public health problem with high associated healthcare costs [2,3]. Cholecystectomy is the primary treatment option for symptomatic GB stones, because 50% of patients have recurring colic [3]. On the other hand, patients with asymptomatic GB stones are not recommended for routine treatment, although approximately 0.7 to 2.5% develop gallstone-related symptoms per annum [3,4]. However, several authors have suggested that asymptomatic and mildly symptomatic patients should be considered potential candidates for gallstone dissolution therapy given a long life expectancy, increased gallstone incidence among young people, and the likelihood of complications [5,6,7]. Tomida et al. reported that asymptomatic GB stone patients that receive bile acid litholysis have significantly lower cumulative probabilities of biliary pain and acute cholecystitis than untreated asymptomatic individuals at 5 and 10 years [7].

The biliary–enteric circulation and the bile acid–gut microbiome axis are closely connected to the biliary system and gut functions [8,9]. Primary bile acid is secreted through the biliary tract to the duodenum, absorbed by the intestinal wall, and re-enters the hepatobiliary system, though a small proportion is converted into secondary bile acids by gut microbiota [8,10]. Abnormalities in the gut microbiome are now considered to contribute to the pathophysiologies of several diseases, including biliary diseases such as malignancies and gallstones [11,12,13,14]. In a previous study, we suggested that duodenal dysbiosis might contribute to common bile duct stone development [11]. Shen et al. identified 13 types of bacteria with potential pathogenic effects in 15 patients with cholelithiasis and suggested that the microbiome could be utilized for the diagnosis and treatment of gallstone disease [12]. Furthermore, Jia et al. reported that two members of the gut microbiome (Lactobacillus and Alloscardovia) might be biomarkers of intrahepatic cholangiocarcinoma [15].

Ursodeoxycholic acid (UDCA) is the 7β-hydroxy isomer of chenodeoxycholic acid (CDCA) and is normally present in human bile at <5% [16]. A synthetic bile acid comprised of UDCA and CDCA has been widely used to treat hepatobiliary diseases, and several studies have shown that UDCA/CDCA-based oral litholysis treatment effectively dissolves gallstones and provides symptom control [5,6,17]. However, bile acids and the gut microbiome are closely related by the bile acid–gut microbiome axis, and changes in bile acid composition caused by the oral administration of UDCA/CDCA probably affect the gut microbial environment. Actually, it was shown in a recent study that gut microbiome dysbiosis in patients with primary biliary cirrhosis might be suppressed by UDCA treatment [18]. However, few studies have addressed the association between UDCA/CDCA administration and the gut microbiome in patients with gallstone disease.

This study was conducted to identify gut microbiome changes induced by UDCA/CDCA administration in patients with GB stones. In addition, we investigated and compared the gut microbiomes of GB stone patients and healthy controls. 

## 2. Methods

### 2.1. Patients and Study Drug

This prospective, single center, preliminary study was performed from May 2021 to April 2022. All patients were recruited from the outpatient clinic of Inha University Hospital (a Korean tertiary referral hospital). The inclusion criteria were: (1) age >18 years, (2) GB stones detected by abdominal ultrasonography, (3) no evidence of a GB stone by plain abdominal radiography, (4) asymptomatic, and (5) UDCA/CDCA naive. The exclusion criteria applied were: (1) complicated GB stones, (2) chronic kidney disease, (3) abnormal liver function test, (4) history of any malignancy, (5) receipt of antibiotic or probiotic therapy within the previous 2 months, (6) symptoms of inflammatory bowel disease or gastrointestinal obstruction, and (7) bacterial diarrhea within the previous 6 months. Healthy controls met the following inclusion criteria: (1) normal range kidney and liver function test results, (2) the absence of hepatitis B/C virus antigen, and (3) no history of malignancy. In addition, those that had taken any probiotic or antibiotic within 2 months of study commencement were also excluded.

This open-label study was conducted using UDCA/CDCA capsules (CNU^®^; Myungmoon Pharmaceutical Company, Seoul, Korea). Each capsule contained 114 mg of UDCA and 114 mg of CDCA, and participants were prescribed two capsules per day (one with breakfast and the other with dinner). The prescribed treatment duration was 6 months.

### 2.2. Study Design

Patients were asked to visit the outpatient clinic 3 and 6 months after treatment commencement. Physical examination and symptom evaluation (presence of nausea, indigestion, diarrhea, and abdominal pain) were performed at baseline and at each visit. Fecal samples were collected at baseline and at 3-month follow-up visits. White blood cell counts (WBCs), C-reactive protein (CRP), total bilirubin (T.Bil), aspartate aminotransferase (AST), alanine aminotransferase (ALT), alkaline phosphatase (ALP), and γ-glutamyl transferase (r-GTP) levels were checked at baseline and 6-month follow-up visits, and abdominal ultrasonography was also performed to evaluate GB stone volume changes. 

### 2.3. The Outcome Definitions

Gallbladder stone volumes were calculated using the radius (r) of the largest gallstone as determined by abdominal ultrasonography, assuming a spherical shape (4/3π × r^3^). Dissolution rates were defined as percentage reductions in GB stone volumes. Complete dissolution was defined as the absence of a GB stone by follow-up abdominal ultrasonography, and partial dissolution was defined as a reduction in gallstone volume of >50%. Response to therapy was defined as complete dissolution or partial dissolution. No meaningful response was defined as a volume reduction of 0 to 50%. 

### 2.4. DNA Extraction from Fecal Samples

Fecal samples were collected before UDCA/CDCA administration and after 3 months of UDCA/CDCA treatment, and microbiome compositions were compared. Samples were stored at −80 °C prior to shipping to Bioeleven Co., Ltd. (Seoul, Republic of Korea) for DNA extraction and sequencing. Samples were initially centrifuged at 5000× *g* for 5 min at room temperature and then resuspended in 500 µL of cetyltrimethylammonium bromide buffer, according to the manufacturer’s instructions. DNA extraction was performed using a Maxwell^®^ RSC PureFood GMO and Authentication Kit (Promega, Madison, WI, USA). Concentrations of bacterial DNA were determined using a UV-vis spectrophotometer (NanoDrop 2000c; Thermo Fisher Scientific, Waltham, MA, USA) and the QuantiFluor^®^ ONE dsDNA System (Promega). All samples were stored at −20 °C until required for further experiments.

### 2.5. PCR Amplification of the V3–V4 Region of the Bacterial 16S Ribosomal RNA (rRNA) Gene

The V3–V4 variable regions of the bacterial 16S rRNA gene were amplified using a two-step PCR protocol. Briefly, PCR was conducted using two primers, F319 (5′-TCGTCGGCAGCGTCAGATGTGTATAAGAGACAGCCTACG-GGNGGCWGCAG) and R806 (5′-GTCTCGTGGGCTCGGAGATGTGTATAAGAGACA-GGACTACHVGGGTATC-TAATCC-3′). PCR products were separated by 2% agarose gel electrophoresis, and 16S rRNA libraries were purified using AMPure XP magnetic beads, according to the manufacturer’s instructions (Beckman Coulter, Wycombe, UK). A Bioanalyzer 2100 (Agilent, Santa Clara, CA, USA) was used to ensure sample purity. For second-round PCR, Illumina Nextera barcodes (Illumina, Inc., San Diego, CA, USA) were attached to first-step PCR products using an i5 forward primer and i7 reverse primer. Amplified products were purified as described for first-round PCR. DNA quantitation was performed using the QuantiFluor^®^ ONE dsDNA System (Promega), and a Bioanalyzer 2100 (Agilent, Santa Clara, CA, USA) was used for sample quality control. Then, 16S rRNA gene amplification and library preparation, which was performed using a two-step PCR protocol, were used to perform 16S rRNA sequencing using a MiSeq v3 Reagent Kit (Illumina, Inc.).

### 2.6. Data Analysis

For data analysis, 16S rRNA sequencing data were processed using the mothur software package (v1.39.4) [19]. Chimeras were detected and removed using Chimera UCHIME. DNA sequences were clustered into operational taxonomic units (OTUs) by reference-based OTU clustering using the SILVA rRNA database (release 102) [20]. Each OTU was assigned taxonomically using the Ribosomal Database Project reference database (training set version 14) [21]. Species richness and differences in microbial profiles were identified using alpha and beta diversities calculated using mothur. Microbial diversities, evaluated using OTU richness, were used to evaluate alpha diversities using Chao1, Shanon, and Simpson indices. Beta diversity provides a measure of compositional dissimilarity between OTUs on phylogenetic trees. Non-metric multidimensional scaling (NMDS) and the phyloseq package for R were used to compare group beta diversities. Linear discriminant analysis with effect size estimation (LEfSe) was conducted to determine intergroup differences in relative abundances of taxa. A linear discriminant analysis score > 2 with a *p*-value < 0.05 was considered statistically significant. Data were visualized using R (version 3.6.0) and the MASS, ggplot2, and reshape2 graphics packages. 

### 2.7. Ethics Statement

The study protocol was approved by the Institutional Review Board of Inha University Hospital (INHAUH 2021-03-051), and written informed consent was obtained from all participants before study commencement. The study was conducted in accordance with the Declaration of Helsinki and Good Clinical Practice guidelines.

## 3. Results

### 3.1. Study Subjects and GB Dissolution Rates after UDCA/CDCA Treatment

Fifteen GB stone patients and 40 healthy controls were initially included. The demographic and clinical characteristics of patients and controls are shown in Table 1. However, two patients dropped out during the 6-month treatment period. Eight of the remaining thirteen patients responded to 6 months of oral UDCA/CDCA treatment; four showed complete stone dissolution, and four partial dissolution. The other five patients showed no meaningful response (*n* = 3) or an increase in GB stone volume (*n* = 2). The overall response rate to UDCA/CDCA treatment was 62%, and the complete dissolution rate was 31% (Table 2). 

### 3.2. Analysis of the Gut Microbiome by 16S rDNA Sequencing

#### 3.2.1. Composition of the Bacterial Communities 

**Figure 1:**
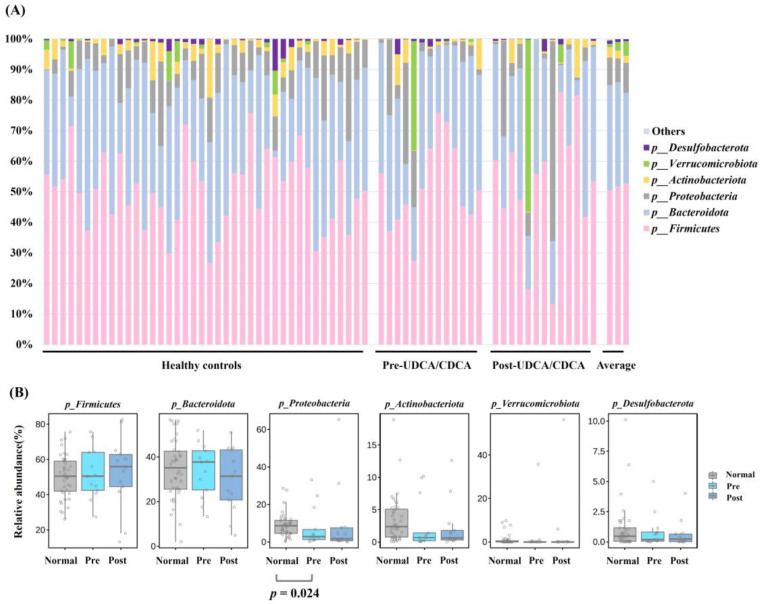
Taxonomic compositions at the major phylum level. (**A**) Bacterial community compositions in the three study groups at the phylum level. (**B**) Significant intergroup differences observed in gut microbial relative abundances at the phylum level.

We analyzed taxonomic compositions and alterations in the gut microbiome in GB stone patients over the 6-month treatment period and compared compositions with those of healthy controls. The compositions and abundances of bacterial communities in the healthy controls and pre- and post-UDCA/CDCA treatment groups at the phylum level are shown in Figure 1A,B. The phyla *Fimicutes*, *Bacteroidota*, *Proteobacteria*, *Actinobacteriota*, *Verrucomicrobiota*, and *Desulfobacteriota* were enriched in all three groups, and, as shown by the figures, relative abundances were similar in the groups. However, aggregate data showed significant differences between gut microbial compositions at the phylum level between the pre-UDCA/CDCA group and healthy controls (Figure 1B). Furthermore, a significantly lower relative abundance of the *Proteobacteria* phylum was observed in the pre-UDCA/CDCA group than in the control group (*p* = 0.024). Phyla that accounted for <1% of microbiomes were designated ‘others’. 

**Figure 2:**
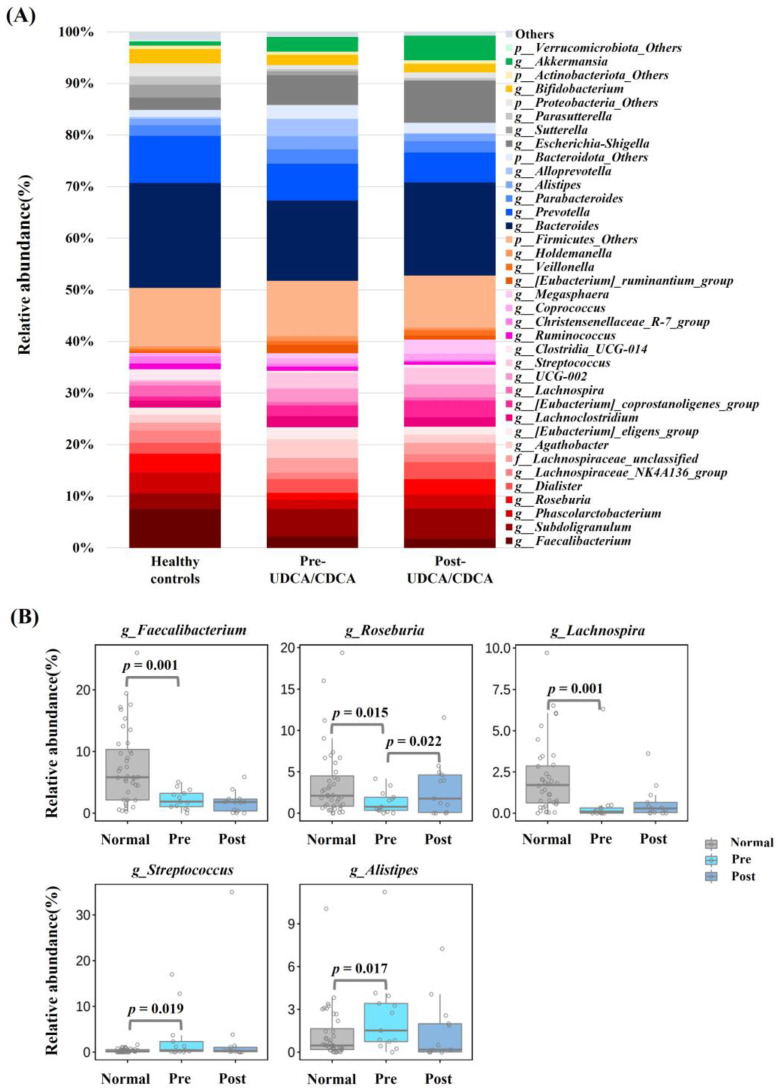
Taxonomic compositions at the genus level. (**A**) Bacterial community compositions in the three study groups at the genus level. (**B**) Significant intergroup differences observed in gut microbial relative abundances at the genus level.

At the genus level, genera with abundances of ≥1% in gut microbiome populations were subjected to statistical analysis, and genera occupying <1% were expressed collectively as ‘others’. Microbiome compositions at the genus level in each group are shown in Figure 2A. The abundances of *Faecalibacterium* (*p* = 0.001), *Roseburia* (*p* = 0.015), and *Lachnospira* (*p* = 0.0001) were lower in the pre-UDCA/CDCA group than in the control group. In contrast, the abundances of *Streptococcus* (*p* = 0.019) and *Alistipes* (*p* = 0.017) were higher in the pre-UDCA/CDCA group.

Interestingly, the abundance of *Roseburia* was lower in the pre-UDCA/CDCA group than in the control group (*p* = 0.015) but recovered in the post-UDCA/CDCA group (*p* = 0.022) to a level similar to that of the control group (Figure 2B).

#### 3.2.2. Microbial Diversity in the Three Study Groups

**Figure 3:**
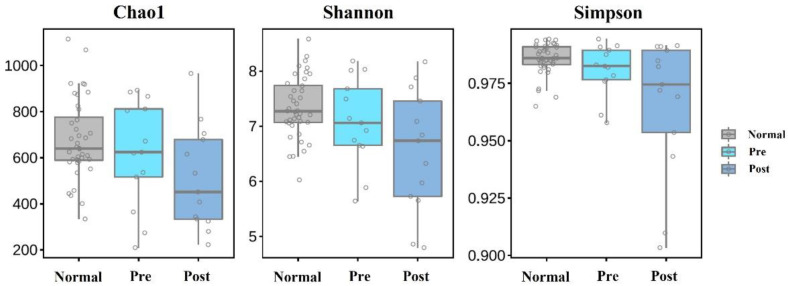
Alpha diversity (Chao1, Shannon, and Simpson indices).

Alpha diversity analysis showed that microbial richness was reduced by UDCA/CDCA treatment. As estimated using Chao1, Shannon, and Simpson indices, gut microbial alpha diversity was similar in the control and pre-UDCA/CDCA groups but significantly lower in the post-UDCA/CDCA group than in the control and pre-UDCA/CDCA groups (*p* < 0.01, *p* < 0.008, and *p* < 0.018, respectively) (Figure 3). 

**Figure 4:**
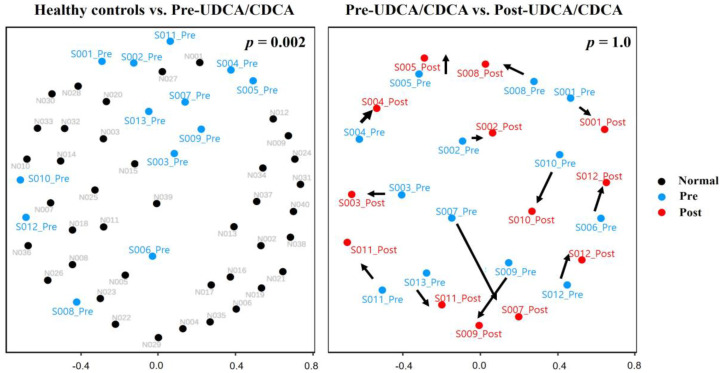
Beta diversity. Non-metric multidimensional scaling (NMDS) ordination of beta diversity analyses at the ASV (amplicon sequence variant) level.

Beta diversity provides a measure of the similarity (or difference) between organismal compositions. Beta diversity was significantly different between pre-UDCA/CDCA group and controls (*p* = 0.002), but no significant difference was observed between the pre- and post-UDCA/CDCA groups (*p* = 1.0) (Figure 4). 

#### 3.2.3. Relative Abundances of Bacterial Communities in the Three Groups

**Figure 5:**
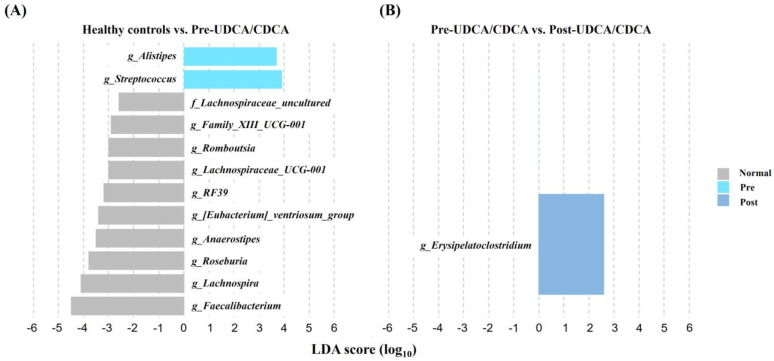
Linear discriminant analysis with effect size (LEfSe) at the genus level. (**A**) Two taxa (*Alistipes* and *Streptococcus*) were enriched in GB stone patients pre-UDCA/CDCA treatment as compared with healthy controls. However, ten taxa including *Faecalibacterium*, *Lachnospira*, and *Roseburia* were more abundant in the healthy control group than in the pre-UDCA/CDCA group. (**B**) Relative abundances of *Erysipelatoclostridium* were significantly higher in the post-UDCA/CDCA treatment group than in the pre-UDCA/CDCA treatment group.

A supervised comparison was conducted by LEfSE to determine which bacteria taxa abundances were significantly changed in GB stone patients by UDCA/CDCA treatment. Two taxa (*Alistipes* and *Streptococcus*) were enriched in the pre-UDCA/CDCA group versus the control group. However, ten taxa, including *Faecalibacterium*, *Lachnospira* and *Roseburia*, were more abundant in the control group than in the pre-UDCA/CDCA group (Figure 5A). Significant microbiome differences between the pre- and post-UDCA/CDCA groups are shown in Figure 5B. Notably, the relative abundance of *Erysipelatoclostridium* was significantly higher in the post-UDCA/CDCA group (*p* = 0.012).

## 4. Discussion

In this study, we analyzed and compared the gut microbiome profiles of GB stone patients before and after 6 months of UDCA/CDCA treatment with those of healthy controls by 16S rRNA sequencing. The gut microbial compositions of patients with GB stones and healthy controls were distinctly different. Furthermore, a comparison of profiles before and after treatment showed that 6 months of UDCA/CDCA treatment significantly modified gut microbiome compositions. To the best of our knowledge, this is the first study to analyze gut microbiome changes before and after UCDA/CDCA treatment in GB stone patients. Although it is difficult to distinguish between the direct and indirect effects of gut microbiota dysbiosis on disease, alterations in gut microbiota have been reported to play important roles in the etiologies of gastrointestinal tract disorders and many non-digestive metabolic diseases, and several biliary tract diseases have also been associated with the gut microbiome [22]. In a previous study, we found that alterations in the bile microbiome might influence the occurrence of GB stones, and the present study presents evidence that the gut microbiome affects the occurrence of GB stones [11,23]. Our findings suggest that alternative therapies are needed that target the gut microbiome in patients with gallstones exhibiting gut dysbiosis. 

Furthermore, our results showed that gut microbial compositions differed in GB stone patients and healthy controls. In particular, the abundances of *Faecalibacterium* (*p* = 0.001), *Roseburia* (*p* = 0.015), and *Lachnospira* (*p* = 0.0001) were lower in GB stone patients than in healthy controls. *Faecalibacterium* is an important component of the healthy human gut microbiome environment and a major source of butyrate, which has an anti-inflammatory function within intestinal mucosa and may help maintain intestinal permeability and gut barrier functions [18]. Several studies have reported that *Faecalibacterium* is significantly more abundant in healthy controls than in patients with biliary tract cancer or GB stone patients [14,24]. In addition, experimental studies indicate that *Roseburia* and *Lachnospira* maintain gut health by selectively stimulating the growth of potentially beneficial gut bacteria and that these bacteria are significantly less abundant in GB stone patients [14,25]. In addition, significant overgrowths of *Streptococcus* and *Alistipes* have been reported in GB stone patients, and both are known to have pro-inflammatory effects and promote gut barrier dysfunction [26,27]. While the precise mechanisms underlying the relationship between gut microbiome and GB stone are still not fully understood, it is clear that the gut microbiome is an important factor to consider in the pathology of GB stones [28,29,30]. In addition to the changes in overall gut microbiota composition, specific gut microbiota lineages have also been implicated in the development and progression of gallstone diseases. Several studies have suggested that dysbiosis or an imbalance of gut microbiota may lead to the release of inflammatory and vasoactive substances as well as changes in bile acid metabolism and host metabolic pathways. These changes may also contribute to the promotion of chronic pro-inflammatory states, as well as the disruption of intestinal permeability [28,31].

Interestingly, we observed that gut microbiome composition at the genus level was altered by UDCA/CDCA treatment and that the abundance of *Roseburia* was significantly lower in the pre-UDCA/CDCA group than in the control group (*p* = 0.022), and restored by UDCA/CDCA treatment to almost the control level. As described above, several studies have suggested that low *Roseburia* abundance is associated with the development of biliary malignancies, which suggests that the restoration of *Roseburia* abundance by UDCA/CDCA has clinical implications for cancer prevention. However, the mechanism responsible for microbiome restoration by UCDA/CDCA treatment has not been elucidated. Nonetheless, a preclinical study reported that UDCA restores gut microbiota balance by supporting the integrities of tight junction proteins in gut epithelium and reducing the expressions of several anti-inflammatory cytokines [32]. Furthermore, it has also been reported that UDCA suppresses apoptosis and stimulates cell survival signaling pathways, and, thus, has a cytoprotective effect [33,34]. Future clinical trials are warranted on the optimal dosage and duration of UDCA/CDCA treatment.

The present study has several limitations that warrant consideration. First, our study was conducted on Korean patients, so caution should be exercised when applying our results to other races and populations with different dietary patterns. Second, although we excluded patients that had taken any type of probiotic or antibiotic during the 8 weeks prior to enrolment, foods and other medications could have triggered gut microbiota dysbiosis and affected bile acid metabolism, and we were not able to control for these potential confounders. Third, multiple comparison adjustments were not applied due to the small sample size and exploratory nature of this study. Fourth, we analyzed microbiota compositions in stool samples, but, given the role played by gut microbiota in bile acid synthesis and metabolism, microbiome and metabolomics analyses of stool samples and blood analysis may have provided additional details on microbiota-related activities in GB stone patients. Fifth, the pathway analysis performed was based on 16S rRNA sequences, and, although 16S rRNA analysis has been commonly used in microbiome research, shotgun sequencing for metagenomic and metatranscriptomic compositions might have provided more precise information on microbial community compositions and functions. Sixth, the roles of bacteria that exhibited abundance differences in responders and non-responders require further study. Finally, the study did not reveal the mechanism underlying changes in gut microbiota composition induced by UDCA/CDCA treatment. Additional studies are needed to elucidate this mechanism and to determine the ability of UDCA/CDCA treatment to prevent biliary malignancies. Nonetheless, the present study indicates that gut microbiome composition abnormalities in GB stone patients may be reduced by UDCA/CDCA treatment. 

In conclusion, the study shows that UDCA/CDCA treatment effectively caused GB stone dissolution and positively impacted gut microbiome composition in GB stone patients. 

## Figures and Tables

**Table 1:** Demographic characteristics of the study subjects.

Variables		GB Stone Patients		Healthy Controls	
	Before UDCA/CDCA (*n* = 13)	After UDCA/CDCA (*n* = 13)	*p* *	(*n* = 40)	*p* *
Age (years) ^§^	61 (40–72)	61 (40–72)		59 (40–77)	0.60
Sex, female, n (%) ^§^	10 (76.9%)	10 (76.9%)		29 (72.5%)	0.75
BMI, kg/m^2 §^	23.5 (21.4–30.8)	23.5 (21.4–30.8)			
WBC (/ul) ^§^	7410 (3560–12,980)	7120 (3750–9090)	0.42		
AST (IU/L) ^§^	23 (16–153)	19 (15–42)	0.15		
ALT (IU/L) ^§^	23 (9–247)	18 (13–39)	0.84		
T.bil (IU/L) ^§^	0.5 (0.3–1.1)	0.4 (0.3–1.1)	0.21		
ALP (IU/L) ^§^	84 (5–224)	67 (1–119)	0.18		
PT (s) ^§^	12.0 (11.4–12.5)	12.3 (11.8–13.3)	0.16		

Abbreviation: BMI, body mass index; WBC, white blood cell count; AST, aspartate aminotransferase; ALT, alanine aminotransferase; T.bil, total bilirubin; ALP, alkaline phosphatase; PT, prothrombin time. ^§^ Median (range). ^*^ *p* values were calculated using the *t*-test or the Chi-square test.

**Table 2:** Responses to UDCA/CDCA dissolution treatment.

Response	Number	%
Complete dissolution	4	31
Partial dissolution	4	31
Subtotal	8	62
No meaningful response	3	23
Increased	2	15
Total	13	100

## Data Availability

The raw data are available from the corresponding author on reasonable request, and planning to be shared in the public repository, NCBI database.
